# Surgical residents’ approach to training: are elements of deliberate practice observed?

**DOI:** 10.12688/mep.19025.1

**Published:** 2022-10-21

**Authors:** Kendra Nelson Ferguson, Josée Paradis

**Affiliations:** 1 Department of Geography, University of Western Ontario, London, Ontario, Canada; 2 Department of Otolaryngology - Head and Neck, University of Western Ontario, London, Ontario, Canada

**Keywords:** deliberate practice; surgical training; medical expertise; surgical residents

## Abstract

**Background:** Research in the area of deliberate practice has consistently shown that intense, concentrated, goal-oriented practice in a focused domain, such as medicine, can improve both skill development and performance to attain a progressively higher standard of excellence. In theory, utilizing deliberate practice in a medical context could result in improved surgical training and in turn better patient outcomes. Therefore, the purpose of this study was to gain a better understanding of how surgical residents approach their training from the perspective of the surgical residents themselves and to explore if elements of deliberate practice are observed.

**Methods:** Eight surgical trainees participated in one of two focus groups depending on their training level (five junior residents; three senior residents). With the exploratory nature of this research, a focus group methodology was utilized.

**Results:** By employing both deductive and inductive thematic analysis techniques, three themes were extracted from the data: learning resources and strategies, role of a junior/senior, and approaching weaknesses.

**Conclusions:** Although elements of deliberate practice were discussed, higher functioning is necessary to achieve performance excellence, leading to improved patient outcomes.

## Introduction

The Royal College of Physicians and Surgeons of Canada has long emphasized the concept of the “competent” physician. Most recently, the Royal College has begun an initiative known as Competency by Design (CBD). The aim of CBD is to improve physician training and enhance lifelong learning by initiating a change from a traditional training model of medical education to a model embracing Competency Based Medical Education (CBME). There are several fundamental differences between a traditional training model and CBME. In contrast to traditional training methods which emphasize learning objectives as outcomes, CBME emphasizes performance outcomes demonstrated by the student ^1^. In addition, the durations of traditional training programs are predetermined, and program completion is reflective of the time-spent within the program. Whereas CBME offers a more flexible time frame and allows residents to progress at varying speeds. Thus, graduation times may differ based on rate of skill acquisition and demonstration of competence. Specifically, CBME focuses on performance outcomes; emphasizes acquired abilities; de-emphasizes time-based training; and is learner centered ^2^.

 In the attainment of excellence, there is a well-known theoretical framework known as deliberate practice ^3^. Deliberate practice encompasses three cognitive processes: preparation and planning, execution of skills, and monitoring of performance ^4, 5^. More specifically the framework advocates that reaching superior performance in one’s domain requires repetitive performance of psychomotor skills (i.e., preparation), proficient problem-solving abilities (i.e., planning), capacity to execute skills smoothly and without effort (i.e., execution of skills), rigorous skill assessment and self-evaluation where weaknesses are identified both from the learner and the educator (i.e., monitoring of performance), and provision of feedback from educators (i.e., monitoring of performance) ^4^. Research in the area of deliberate practice consistently shows that intense, concentrated practice in a focused domain can provide the best conditions for performance improvement in contrast to innate abilities ^4– 6^. Further, learners must pursue independent practice beyond routine behaviour to achieve real expertise ^4^.

Deliberate practice has mostly been studied in music, sports, and chess. Ericsson *et al.* ^3^ showed that in expert performers (i.e., musicians, athletes, chess players), there is no correlation between level of expertise and hours spent practicing. However, the number of hours spent in deliberate practice was positively correlated to level of expertise. Medical educators are recognizing the value of examining deliberate practice within the realm of medical education. Moulaert *et al*. ^7^ investigated the relationship between aspects of deliberate practice and study achievements among undergraduate medical students ( *N* = 777). Results indicated that the “high achieving” students exhibited more elements of deliberate practice than “low achieving” students ^7^. Wayne *et al*. ^8^ designed an educational intervention featuring deliberate practice with third-year residents ( *N* = 40) in an internal medicine residency program. It was found that the structured educational program offering an opportunity for deliberate practice produced large and consistent improvements in residents' skills. Additionally, Duvivier *et al*. ^9^ examined the role of deliberate practice in medical students’ ( *N* = 875) development of preclinical skills training. Clinical skills scores correlated with sub-scores, reflecting the use of deliberate practice.

Overall, the very nature of deliberate practice focuses on the trainee’s abilities and at its core is learner centered, with the ultimate end goal of achieving expert performance, which in theory would result in improved patient outcomes. Therefore, the notion of deliberate practice embodies many aspects of CBME. To date, little is known about how residents approach their surgical training, how they evaluate their current weaknesses and develop a pathway to address these, and how they plan to transition from one milestone to another. Without knowledge of residents’ role in their development, educators miss the opportunity to optimize progression of these lifelong learning skills. Therefore, the purpose of this study was to gain a better understanding of how surgical residents approach their surgical training from the perspective of the surgical residents themselves and to explore if elements of deliberate practice are observed.

## Method

### Ethics and consent

Study approval was obtained from Western University’s health sciences research ethics board (#108993; June 2017). Participants provided written informed consent prior to the focus groups beginning.

### Study design

 A qualitative approach was adopted, consisting of two focus group discussions with junior surgical residents (defined as traditional surgical training years PGY1-3) and senior surgical residents (defined as traditional surgical training years PGY4-5). Grouping by resident training level is important as junior and senior residents may approach their training differently based on level of performance. Using focus groups allowed researchers to gain insight into participants’ experiences, attitudes, and perspectives about the topic being discussed. With the exploratory nature of this research, a focus group methodology seemed appropriate, allowing researchers to gain insight into the topic while revealing relevant trajectories in furthering exploration of this topic ^10^.

### Participants

 Participants were recruited September 2017 and purposively sampled from a surgical training program in Southwestern Ontario, specifically one of the first surgical programs in Canada to adopt the CBME approach. Surgical residents were identified by surgical program directors from the corresponding training program and then contacted via email. Potential participants were provided with a letter of information detailing the purpose of the study, information on confidentiality, and the use of data. Participants were asked to confirm if they were able and willing to participate in each focus group discussion. A schedule poll was sent to prospective participants to determine the best date and time to schedule each group. Therefore, the first focus group consisted of five junior residents from obstetrics/gynecology, otolaryngology, and general surgery (2 male, 3 female) and the second focus group included three senior residents from obstetrics/gynecology and otolaryngology (2 male, 1 female). Both focus groups took place in October 2017. There was no non-participation.

### Data collection

The second author (MD, pediatric otolaryngologist, female) in accordance to the theoretical framework of deliberate practice ^3^. The questions focused on elements of deliberate practice such as: preparation and planning (e.g., remember the last time you were in the operating room, how did you prepare/plan for the surgical case?), reflection of self-assessed skills (e.g., strengths and weaknesses), goal setting (e.g., prior to scrubbing into the case, what specific surgical goals did you have in mind?), the use of observation and imagery (e.g., how do you use observation and/or imagery?), self-evaluation (e.g., intra-operatively, how do you self-evaluate your own skills and against what standard?), and seeking feedback (e.g., do you ask for feedback or do you wait to see if it is given?).

Focus groups took place in a meeting room at the affiliated hospital. Prior to the start of each focus group, participants were informed that data will be used anonymously and were asked to sign a consent form, agreeing to an audio recording of the discussion and data to be used for publication purposes. The same guide was followed for each group ^11^. Participants were seated at a round table to allow for face-to-face contact and a brief demographic questionnaire was administered. Further, participants were provided with an explanation of the research goal and were assured that the information collected will not be identifiable or used for purposes other than research. Participants were also informed that they could skip any question they wished not to answer. Each focus group included a facilitator (i.e., second author). Although the facilitator is a practicing otolaryngologist in the same hospital as the participants, they were not involved in any of the participants training or education and acted as a neutral outsider interested in participant’s experiences ^11^. The facilitator actively generated interaction and discussion between participants with specific probes. Data saturation was considered once no new information emerged from the participants during data collection. The total duration of the junior group discussion was 62.5 minutes and the senior group discussion lasted 88 minutes.

### Data analysis

 Each interview was audio-recorded and transcribed verbatim by the first author (PhD, female) resulting in 72 pages of transcripts. The second author then reviewed audio-recordings and transcripts to ensure accuracy. Following this, transcripts were sent to participants for review and verification prior to data analysis. No modifications and/or additions were made by participants. The first author immersed themselves in the data by reading and re-reading the transcripts to fully comprehend the content of interactions. A deductive and inductive thematic approach was used to categorize responses from participants. This approach allowed for components of theory (i.e., deliberate practice) to guide the deductive thematic analysis while permitting other themes to emerge directly from the data using inductive coding ^12^. Following the deductive analysis, an inductive approach was used to finalize themes and sub-themes from each group ^12^. Overarching categories were then created to represent each theme and sub-theme. Categories, themes, and sub-themes were sorted, compared, and contrasted in each age cohort to uncover any differences. The second author then independently analyzed the data and derived a set of themes and sub-themes from the discussions. Both authors compared analyses to ensure validity. Disagreements on themes and sub-themes were resolved through discussion. A percentage agreement greater than 85% was achieved, which is considered “good”, and thus the coding process can be deemed reliable ^13^. The second author, who conducted each focus group, acknowledged that this position could affect the research process and outcome. Therefore, recommendations provided by Berger ^14^ were followed in the form of reflexivity to monitor involvement and detachment of the researcher and the researched as a means to enhance the rigor of the findings. 

## Results

 Data collected from both junior and senior focus groups is presented simultaneously. Table 1 (i.e., junior participants) and Table 2 (i.e., senior participants) illustrate a breakdown of categories, themes, and sub-themes. Sub-themes are explored under each category and theme heading. By presenting verbatim quotes in focus groups research, the reader can gain insight into participant interactions and the constructs that arose ^15^. Codes have been used to maintain participants’ anonymity.

**Table 1.** Junior data: categories, themes, and sub-themes with number of responses.

Categories	Themes	Sub-themes (number or responses)
Methods of preparation	Learning resources and strategies	Online video resources (3/5) Books/textbooks (4/5) Reviewing charts (3/5) Imagery (3/5)
Intra-operative role	Role of a junior	Observing and assisting (3/5)
Intra-operative weaknesses	Approaching weaknesses	Fear of exposing weaknesses (3/5)

**Table 2.** Senior data: categories, themes, and sub-themes with number of responses.

Categories	Themes	Sub-themes (number or responses)
Methods of preparation	Learning resources and strategies	Online video resources (3/3) Books/textbooks (2/3) Reviewing charts (2/3) Imagery (3/3)
Intra-operative role	Role of a senior	Educator (3/3)
Intra-operative weaknesses	Approaching weaknesses	Open communication with staff (3/3)

### Preparation: Learning resources and strategies

 Junior and senior residents discussed their methods of preparation for surgical cases and specific procedures. Both groups identified four sub-themes: online video resources, books/textbooks, reviewing charts, and the use of imagery.

***Online video resources.*** When exploring data from the junior focus group, it was apparent that online video resources allowed for a more accurate depiction of anatomy, surgical technique, and general overviews of procedures. One participant stated: ‘I like videos. I’ve found that repetition is really important in my learning, you can pause and rewind’ (J3). The same participant continued by saying, ‘I like seeing live anatomy better than textbook anatomy as you can see how it is in relation to 3D space. Generally, I watch the anatomy first then surgical technique’ (J3). Another participant mentioned how they ‘typically start with microsurgeon.org as it gives a very brief overview of the anatomy and procedures’ (J2). All senior participants unanimously agreed that online video resources aid in preparation for specific procedures. S1 said, ‘YouTube is a good resource when you're studying or you're in clinic and there are certain manoeuvres or tests that you want to see how it's done. There's a lot of good videos to help’. Relatedly, another participant added that ‘the benefit is you can watch it multiple times. Whereas in an OR case, if you miss something, it's gone like you can’t pause’ (S2). Lastly, it was added that videos help to ‘mentally prepare for the approach that [is] going to [be] taken and the tools used’ (S3).

***Books and textbooks.*** It was evident that most of the junior and senior residents found books and textbooks to be a useful learning resource. As mentioned by one junior participant, ‘I read beforehand of the major kinds of things that I would see like the more common things right now as a junior, so the differentials of dizziness, vertigo, and then going through sign and symptoms of each one’ (J4). Comparably, another junior resident said, ‘[I] tend to review the chapter in Cummings that relates to the case so a patient with a unilateral nasal mass, just kind of reviewing and running through sinus surgery briefly in terms of important landmarks and structures’ (J1). It was also noted that reviewing anatomy helps with understanding certain approaches: ‘The night before I read up on anatomy, first and foremost to know the approach’ (J5). Similarly, one senior resident expressed, ‘for certain types of treatments that I haven't seen in a long time, I read about the disease process, so I have a bit more background’ (S2). Another senior resident discussed that they do ‘about 10-15 minutes of reading on the different types and ways to repair or what we might do’ (S1) prior to a procedure.

***Reviewing charts.*** Some of the participants in the junior focus group conversed about how reviewing patient charts to prepare. This can be captured in the subsequent quote: ‘I review the chart, usually power chart and a lot of patients especially in head and neck will have pre-anaesthesia and pre-medicine which is good to get a summary of everything’ (J4). Another junior participant mentioned that they ‘[review the] patient’s chart [that has] been admitted to know their history but also to review the console notes like medications and anaesthesia’ (J2). For seniors, reviewing charts was also an important preparation strategy for most participants. As stated by S1, ‘it's important to look at the patient's charts ahead of time and review the potential post-operative course if I'm not familiar with it’. S2 also said, ‘[I] go through power chart. Read up on the cases the day before and read about their past medical’.

***Imagery.*** Most of the junior residents considered imagery to be a helpful technique when preparing for specific surgical procedures. This can be comprehended in the following quote: ‘I’ll usually go through the equipment and in doing that I go through the steps in my head of what we're doing next’ (J4). They went on to say, ‘I set everything up in order and I’m like OK first we do this then we use the needle and so on’ (J4). J2 also said, ‘I typically prepare by going through steps in my mind so if I was asked to do it like I would at least be able to make an attempt’. Imagery was also a significant preparation strategy for senior participants. One participant said, ‘I go into the actual steps, how I'm going to position myself, how high I want the bed, what I'm going to do, what instruments I need, so I mentally rehearse the operation and how I see it going’ (S1). Another participant expressed, ‘[If] I’m not 100% comfortable before going in, I’ll picture what the steps are in my head like actual anatomy not from a textbook but what I've seen and say this is what I have to do first, etcetera’ (S2). Based on the dialogue in both groups, imagery can assist with preparing for what the surgery may look like and how it can be executed.

### Intra-operative role: Role of a junior vs. role of a senior

 Each group communicated about their intra-operative role as surgical residents. From the conversations had, it was clear that each group viewed their role in the operating room differently. Juniors identified one sub-theme in relation to their intra-operative role: observing and assisting. Moreover, one sub-theme emerged from the analysis of the senior focus group: educator.

***Observing and assisting.*** Most participants in the junior group agreed that their primary role is to observe and assist when needed. One participant mentioned, ‘I think it's hard to know what your role is as a junior so I think right now the most I can do is just try to observe and understand what's going on in the surgery and learn as much as I can’ (J4). Another participant interjected by saying, ‘there’s a fellow on right now and he's primarily going to do all the flaps so [I’ll] assist and observe if he needs help’ (J2). The exchange finished with J5 talking about how observing positions of patients and certain techniques in the operating room may lead to aiding in the procedure: ‘Focusing on what position the patient should be in and how to prepare the patient for initial things like incisions and flaps may lead to me participating in the procedure’.

***Educator.*** For seniors, participants discussed their role as educator. This can be emphasized in the following quote: ‘The goal is to be able to provide juniors with hands on experience. It requires me to be more comfortable with the skills myself so then I can supervise juniors and provide guidance around cases’ (S3). In addition, S1 said, ‘a junior resident is great to have around because you can get them ready for a case beforehand and you can teach a case and what the steps are going to be. It solidifies your knowledge’. As dialogue around being educators continued, seniors acknowledged that this role allowed for more confidence and comfort when performing surgical procedures.

### Intra-operative weaknesses: Approaching weaknesses

 Both junior and senior residents conferred about approaching intra-operative weaknesses. Interestingly, junior residents disclosed their fear of exposing weaknesses, whereas seniors revealed the importance of maintaining open communication with staff.

***Fear of exposing weaknesses.*** Junior residents engaged in conversation about their fears of exposing weaknesses. One participant said, ‘I think certain people may be afraid of showing weaknesses in front of staff. You don’t want to take away your shot at trying something. I think that’s the hesitancy about asking staff for help’ (J3). Comparably, J1 stated, ‘you don’t want things getting taken away from you like maybe you feel uncomfortable with one procedure, which is technically more junior than something else you've been allowed to do but you don't want to come back to second base’. Participants considered how exposing fears, mistakes, and discomforts may result in less opportunity.

***Open communication with staff.*** Senior residents acknowledged the importance of keeping open communication with staff. This can be captured in the following quote: ‘I ask staff when I'm struggling. You know like how do you do this and how come I’m not getting the angle that you’re getting’ (S3). Similarly, S2 said, ‘if I end up struggling, I’ll say at the time to staff this is the part that I always have trouble with like I’ll just say it in the OR and ask for assistance’. Lastly, S1 stated, ‘I have no problem asking for assistance but when it's something that I know is straightforward I like being able to struggle a bit on my own before asking’.

## Discussion

The aim of this study was to gain a better understanding of how surgical residents approach their training. Further, the purpose was to explore if elements of deliberate practice are present in residents training and preparation prior to surgical cases. In fields demanding consistent superior performance, such as medicine, utilizing elements of deliberate practice is critical in achieving expert performance ^4– 6^, and in turn improving patient outcomes.

Examining whether residents deliberately approach their surgical training required investigation into trainees’ cognitive processes (i.e., preparation and planning, execution of skills, monitoring of performance) ^4, 5^. Of these processes, only two were identified: preparation and planning, and execution. Although these processes were recognized, they were discussed with hesitation. Within the element of preparation and planning, participants discussed the various learning resources used to prior for surgical cases (online video resources, books/textbooks, reviewing charts, imagery). Junior and senior residents used similar preparation approaches, however, it was evident that juniors were focused on learning and memorizing anatomy and/or surgical steps, whereas seniors focused on reviewing processes and mental preparation such as imagery.

In terms of execution, participants discussed their intra-operative roles, which differed between junior and senior residents. For juniors, participants felt that their role was to observe and assist if/when prompted. Senior residents viewed themselves as educators, providing juniors with intra-operative experience, knowledge, and guidance. Consistent with early research ^9^, results indicate that moving through residency and gaining hands on experience provides the skill and confidence to guide those in years behind them. Interestingly, when residents were prompted about their use of goal setting, goals focused on preparedness rather than competency. Setting well-defined, deliberate goals with higher performance standards can excel skill execution ^3, 4, 16^.

 The third process (i.e., monitoring of performance) relates to a trainee’s ability to self-evaluate or assess their skills ^4^. It became apparent that this cognitive process was missing from participant’s practice. The development and refinement of psychomotor skills can be largely attributed to a trainee’s ability to evaluate their performance ^4, 5^. Independent self-evaluation can be particularly challenging in the initial years of residency (i.e., year 1 and 2), therefore educators need to ensure that evaluation techniques are used for learning purposes, and not just evaluation purposes ^17– 19^.

Another critical component of performance monitoring is feedback. Although participants did not directly discuss whether they sought or welcomed feedback, they did converse about ways in which they approached intra-operative weaknesses. Methods varied between junior and senior residents. Juniors emphasized fear of exposing weaknesses with hesitation in asking for guidance or feedback from their superior(s). This may point to the need for a better designed learning environment for trainees to feel comfortable in actively seeking feedback ^20, 21^. In contrast, senior residents sought help and advice for skill development, evaluation, and/or general assistance during surgical procedures to improve competence and performance. It should be noted that deliberate practice relies on the learner’s ability to recognize performance weaknesses and take measures to address them, which can be acquired through observation and feedback ^5, 9^. Research has shown that expert performance develops gradually, with performance improving as education, training, and practice advances ^5^. Therefore, as trainees move through their surgical residency program, they may feel more confident in directing attention on improvable aspects ^5^.

Research has shown that deliberate practice may be a tool for improving practice and training, however, there are some limitations to implementing such practices into surgical training programs. Surgical training is challenged by an increase in demand for efficiency in the operating room and the multiple responsibilities residents must carry out ^1, 22^. Both have been shown to contribute to fewer opportunities for teaching and learning, making teacher-guided practice harder to achieve ^5, 23, 24^. These constraints warrant further investigation as it is critical for educators to create training opportunities for deliberate practice that is appropriate for skill development and improvement. These teachable moments allow performers (i.e., surgical residents) to learn and adjust specific aspects of performance to effectively integrate them into practice ^16^.

Future research is required to determine sufficient strategies and techniques for trainees and educators to implement deliberate practice during surgical residency. Further research is necessary to investigate if surgical fellows and staff surgeons are more likely to use imagery as a form of mental preparation when compared to more novice learners. In addition, the development and distribution of a tool to measure deliberate practice is necessary to optimize education, training, and practice.

While this study is exploratory and did not include a large sample, these results are surprising as surgeons bear responsibility for the health and wellbeing of their patients. Findings suggest that residents need to be more deliberate in their practice. To attain expert performance, proficiency must extend beyond experience, general education, and domain-related knowledge ^4, 16, 25^. Higher functioning in the acquisition of planning, execution, monitoring, and evaluation of performance is critical to reach expert performance.

## Data Availability

Transcripts cannot be made publicly available due to ethical policies at the institutional level. Upon request of the lead author, Dr. Kendra Nelson Ferguson ( knelso42@uwo.ca), a detailed data summary can be provided with all participant quotes in accordance with themes and sub-themes. Applicants must be bona fide researchers and/or medical professionals. figshare: Interview Guide - Focus Group- Phase 1.docx. https://doi.org/10.6084/m9.figshare.21346830.v1 ^26^ Data are available under the terms of the Creative Commons Attribution 4.0 International license (CC-BY 4.0).
