# Supplementary material for: Surgical residents’ approach to training: are elements of deliberate practice observed?
Source: MedEdPublish (2016). 2022 Dec 2;12:62. Originally published 2022 Oct 21. [Version 2] doi: 10.12688/mep.19025.2 (PMC10818099; doi:10.12688/mep.19025.2)
Supplement: Peer review history

# Reviewer report: Reviewer response for version 2

**DOI:** 10.21956/mep.20843.r32809

**Published:** 2024-01-26

**Authors:** Anne M. Messman, Urvashi Katiyar

**Affiliations:** 1 Vice Chair of Education, Department of Emergency Medicine, and Associate Dean of Graduate Medical Education, Wayne State University School of Medicine, Detroit, MI, USA; 2 Sinai-Grace Hospital, Detroit, MI, USA

No revisions were made to the background section of the abstract so I continue to find it confusing.

Have any limitations of the research been acknowledged?

Yes

Is the work clearly and accurately presented and does it cite the current literature?

Yes

If applicable, is the statistical analysis and its interpretation appropriate?

Partly

Are all the source data underlying the results available to ensure full reproducibility?

Yes

Is the study design appropriate and is the work technically sound?

Partly

Are the conclusions drawn adequately supported by the results?

Partly

Are sufficient details of methods and analysis provided to allow replication by others?

Yes

Reviewer Expertise:

Medical education research including a Master of Health Professions Education from the University of Michigan and AAMC certification in Medical Education Research (in addition to being a practicing MD in the field of emergency medicine).

We confirm that we have read this submission and believe that we have an appropriate level of expertise to confirm that it is of an acceptable scientific standard.

# Reviewer report: Reviewer response for version 2

**DOI:** 10.21956/mep.20843.r32810

**Published:** 2022-12-16

**Authors:** Subha Ramani

**Affiliations:** 1 Division of General Internal Medicine and Primary Care, Department of Medicine, Brigham and Women's Hospital, Boston, MA, USA

Nothing else to add.

Have any limitations of the research been acknowledged?

No

Is the work clearly and accurately presented and does it cite the current literature?

Yes

If applicable, is the statistical analysis and its interpretation appropriate?

Yes

Are all the source data underlying the results available to ensure full reproducibility?

Partly

Is the study design appropriate and is the work technically sound?

Yes

Are the conclusions drawn adequately supported by the results?

Partly

Are sufficient details of methods and analysis provided to allow replication by others?

Yes

Reviewer Expertise:

Qualitative research

I confirm that I have read this submission and believe that I have an appropriate level of expertise to confirm that it is of an acceptable scientific standard.

# Reviewer report: Reviewer response for version 1

**DOI:** 10.21956/mep.20383.r32732

**Published:** 2022-11-22

**Authors:** Subha Ramani

**Affiliations:** 1 Division of General Internal Medicine and Primary Care, Department of Medicine, Brigham and Women's Hospital, Boston, MA, USA

The paper is well written, well referenced and the authors have emphasized the importance of deliberate practice in the acquisition of surgical skills.

 The research questions are addressed appropriately by qualitative study design.

 The introduction discusses deliberate practice in detail, definition, the process, the steps and its importance. However, the problem statement could be better delineated- what is the educational problem and why is it important to study. The gap in literature is discussed briefly in the last para of the intro - I wonder if there was a way to bring it in earlier. It leads to a clear study aim/purpose.

 The methods section is well written. One aspect about choice of focus groups is how group interactions enrich the narratives. This is not discussed. Focus groups may be a group of individuals, but how their narratives are enriched by the group is important. Exploratory nature of research could also use observations or one on one interviews. Some experts may also comment that a focus group of 3 may be rather small. 

 As authors have acknowledged, the sample is small and saturation may be impossible to achieve with this number.

 The first sentence under data collection seems incomplete. Otherwise, data collection and analysis are discussed in detail.

 Reflexivity is not discussed and this is an important concept for qualitative research.

 Results section is reasonably well laid out with categories, themes and sub-themes. Perhaps it could be more succinct and organized, as the authors' description of the theme followed by representative quotes. Since there are many quotes in text, some sections may be hard to follow for readers.

 Discussion follows the steps of deliberate practice and the authors have suggested gaps based on their residents' narratives. For example, they suggest that self-reflection and seeking feedback were not emphasized in the narratives. These are useful data for trainers and educational leaders.

 I do not see a clear limitations section except a mention of small numbers. There are many other methodologic limitations that need to be described.

 Implications of these findings for their own institution and for surgical educators elsewhere could also be outlined in greater detail."

Have any limitations of the research been acknowledged?

No

Is the work clearly and accurately presented and does it cite the current literature?

Yes

If applicable, is the statistical analysis and its interpretation appropriate?

Yes

Are all the source data underlying the results available to ensure full reproducibility?

Partly

Is the study design appropriate and is the work technically sound?

Yes

Are the conclusions drawn adequately supported by the results?

Partly

Are sufficient details of methods and analysis provided to allow replication by others?

Yes

Reviewer Expertise:

Qualitative research

I confirm that I have read this submission and believe that I have an appropriate level of expertise to confirm that it is of an acceptable scientific standard, however I have significant reservations, as outlined above.

# Response

**Published:** 2022-11-25

**Authors:** Kendra Nelson Ferguson

**Affiliations:** University of Western Ontario, Canada

Dear Dr. Ramani,  Thank you for reviewing this manuscript. Your suggestions and feedback have helped strengthen the paper, and I am very appreciative of your time. I have included your comments/suggestions and have responded to them in **bold** below. 

 . The introduction discusses deliberate practice in detail, definition, the process, the steps and its importance. However, the problem statement could be better delineated- what is the educational problem and why is it important to study. The gap in literature is discussed briefly in the last para of the intro - I wonder if there was a way to bring it in earlier. It leads to a clear study aim/purpose.

- **With the nature of the background, literature review, and purpose statement, we wanted to ensure we were building the paper accurately to inform the reader. Although the gap in literature is stated in the last paragraph, we think it builds a suitable rationale before moving on to the methods section.**

2. The methods section is well written. One aspect about choice of focus groups is how group interactions enrich the narratives. This is not discussed. Focus groups may be a group of individuals, but how their narratives are enriched by the group is important. Exploratory nature of research could also use observations or one on one interviews. Some experts may also comment that a focus group of 3 may be rather small.

- **To this point, the following was added to the study design section: “** **Using focus groups allowed researchers to gain insight into participants’ experiences, attitudes, and perspectives through meaningful interactions and narratives, enriching the nature of the data** **^10^** **^”^.** 

3. The first sentence under data collection seems incomplete. Otherwise, data collection and analysis are discussed in detail.

- **The first sentence was revised to: A semi-structured interview guide was developed** **in accordance to the theoretical framework of deliberate practice ^3^ by the second author (M.D.).**

4. Reflexivity is not discussed and this is an important concept for qualitative research.

- **There is a statement in the data analysis section pertaining to reflexivity: “The second author, who conducted each focus group, acknowledged that this position could affect the research process and outcome. Therefore, recommendations provided by Berger** **^14^** **were followed in the form of reflexivity to monitor involvement and detachment of the researcher and the researched as a means to enhance the rigor of the findings”.**  

5. Results section is reasonably well laid out with categories, themes and sub-themes. Perhaps it could be more succinct and organized, as the authors' description of the theme followed by representative quotes. Since there are many quotes in text, some sections may be hard to follow for readers.

- **We appreciate the thought here. We have left the results section as is as we feel the integration of quotes throughout help to bring attention to each theme and subtheme.**

 6. I do not see a clear limitations section except a mention of small numbers. There are many other methodologic limitations that need to be described.

- **A limitations section was added: “** **The findings from this research have important implications, but it is not without limitations. Given the small and heterogenous sample, results cannot be generalized to surgical residents beyond this sample. Additionally, variables such as sex and gender were not accounted for, limiting the scope of the research. As surgical residents were grouped based on program year and evaluations of residents’ skills were not conducted, we are not able to determine if higher performing residents were using more deliberate practice than their lower-performing counterparts”.**

7. Implications of these findings for their own institution and for surgical educators elsewhere could also be outlined in greater detail.

- **Although implications are indirectly mentioned throughout the discussion, the following has been added as concluding sentence: “** **Implications of these findings further highlight the importance of training institutions and surgical educators in facilitating skill development by equipping trainees with the skills necessary to use deliberate practice in their attainment of excellence. Surgical programs could benefit from emphasizing a learning environment that fosters active participation, where trainees feel motivated and comfortable to seek guidance and advice across their novice-expert continuum”.**
- **In addition to your feedback and suggestions, I have removed the section on future research from the discussion and have added the suggestions to the appropriate paragraphs throughout the discussion for better readability and purpose. **​​​​​​​

# Reviewer report: Reviewer response for version 1

**DOI:** 10.21956/mep.20383.r32682

**Published:** 2022-11-02

**Authors:** Anne M. Messman, Urvashi Katiyar

**Affiliations:** 1 Vice Chair of Education, Department of Emergency Medicine, and Associate Dean of Graduate Medical Education, Wayne State University School of Medicine, Detroit, MI, USA; 2 Sinai-Grace Hospital, Detroit, MI, USA

Generally speaking, the article explores whether surgical residents are using elements of deliberate practice during their training and the authors describe some elements that the residents use, but it's not clear why this is important. They are basically asking a very small, heterogeneous group of residents from different specialties to describe how they learn/prepare for cases, then seeing if they are already using deliberate practice. What would be much more interesting would be to see if the higher performing residents are using deliberate practice more than their lower-performing counterparts, or if teaching the residents how to consciously use deliberate practice results in better performance.

 Abstract

- The background is a little confusing in terms of explaining the purpose of the study and how deliberate practice factors into the study. Given that readers are often going to decide whether or not to read the full article based on the abstract, this should be made more clear.

Introduction

- Good explanation of the history of CBME and deliberate practice and their use in medical education. Although these explanations are clear and interesting, the authors could do a better job of linking these concepts to the purpose of their study. It seems that the entire discussion of CBME could be removed without adversely affecting the explanation of the study.
- The mention of the study by Duvivier et al. at the end of the second-to-last paragraph is confusing. What are the sub-scores that the authors reference?

Methods

- I think it is interesting that the authors chose to divide the focus groups by PGY level. I'm not sure that I understand why there would be a difference between these two groups. Would it make more sense to divide the focus groups into those that are higher achieving and those that are not and see if the higher achieving group uses deliberate practice more often? That would support the authors' assertion that deliberate practice is helpful to the learning process.
- Why collect/report gender information if it has nothing to do with the stated purpose of the study?
- The first sentence in the section "Data collection" does not make sense.
- The questions asked of the focus group participants feel like they are leading. For example, asking "how did you prepare/plan for the surgical case" assumes that the learner did some sort of preparation/planning. It would be less leading to ask "did you prepare/plan for the surgical case" and then explore further if the answer is "yes".
- How did the authors decide which questions to ask during the qualitative interview? Do any of the authors have advanced training on qualitative interviewing and analysis of qualitative data?

Results

- Clearly presented.

Discussion

- Although the ultimate goal is to improve patient outcomes, the authors did not study this so they should get rid of the phrase "...and in turn improving patient outcomes" at the end of the first paragraph.

Have any limitations of the research been acknowledged?

Yes

Is the work clearly and accurately presented and does it cite the current literature?

Yes

If applicable, is the statistical analysis and its interpretation appropriate?

Partly

Are all the source data underlying the results available to ensure full reproducibility?

Yes

Is the study design appropriate and is the work technically sound?

Partly

Are the conclusions drawn adequately supported by the results?

Partly

Are sufficient details of methods and analysis provided to allow replication by others?

Yes

Reviewer Expertise:

Medical education research including a Master of Health Professions Education from the University of Michigan and AAMC certification in Medical Education Research (in addition to being a practicing MD in the field of emergency medicine).

We confirm that we have read this submission and believe that we have an appropriate level of expertise to confirm that it is of an acceptable scientific standard, however we have significant reservations, as outlined above.

# Response

**Published:** 2022-11-25

**Authors:** Kendra Nelson Ferguson

**Affiliations:** University of Western Ontario, Canada

Dear Dr. Messmar and Dr. Katiyar, Thank you kindly for your time and efforts with this review. Your feedback and suggestions have helped strengthen this manuscript. I have provided your comments/suggestions and have responded to each suggestion in **bold** below. 

 1. Generally speaking, the article explores whether surgical residents are using elements of deliberate practice during their training and the authors describe some elements that the residents use, but it's not clear why this is important. They are basically asking a very small, heterogeneous group of residents from different specialties to describe how they learn/prepare for cases, then seeing if they are already using deliberate practice. What would be much more interesting would be to see if the higher performing residents are using deliberate practice more than their lower-performing counterparts, or if teaching the residents how to consciously use deliberate practice results in better performance.

- **To comment on your general statement above (i.e., what would be much more interesting would be to see if the higher performing residents are using deliberate practice more than their lower-performing counterparts, or if teaching the residents how to consciously use deliberate practice results in better performance), we were not ethically allowed to group participants as “higher performers” or “lower performers”, however, we have developed a scale that is currently being validated to better understand performance differences across surgical residents.**

**2. Abstract - **The background is a little confusing in terms of explaining the purpose of the study and how deliberate practice factors into the study. Given that readers are often going to decide whether or not to read the full article based on the abstract, this should be made more clear.  

- **To enhance clarity of our background statement, it has been revised to: “** **Deliberate practice research has consistently shown that intense, concentrated, goal-oriented practice in a focused domain, such as medicine, can improve skill development and performance. To date, little is known about how surgical residents approach their surgical training, how they evaluate their current weaknesses, and how they plan to transition from one milestone to another. Without knowledge of residents’ role in their development, educators miss the opportunity to optimize progression of these lifelong learning skills. Therefore, the purpose of this study was to gain a better understanding of how surgical residents approach their surgical training from the perspective of the surgical residents themselves and to explore if elements of deliberate practice are observed”.**

3. Introduction - Good explanation of the history of CBME and deliberate practice and their use in medical education. Although these explanations are clear and interesting, the authors could do a better job of linking these concepts to the purpose of their study. It seems that the entire discussion of CBME could be removed without adversely affecting the explanation of the study.

- **The purpose of this first paragraph is to showcase the similarities between the recent shift to CBME and deliberate practice. As noted in the following sentence, which has been revised to include the overlap: “** **Overall, the very nature of deliberate practice focuses on the trainee’s abilities and at its core is learner centered, with the ultimate end goal of achieving expert performance. Therefore, the notion of deliberate practice embodies many aspects of CBME (i.e., performance outcomes, acquired abilities, learner centered)”.**

4. The mention of the study by Duvivier et al. at the end of the second-to-last paragraph is confusing. What are the sub-scores that the authors reference?

- **This sentence has been revised to:** **Clinical skills scores positively reflected the use of deliberate practice, where students showed improvement and refinement in their performance.**

5. Methods - I think it is interesting that the authors chose to divide the focus groups by PGY level. I'm not sure that I understand why there would be a difference between these two groups. Would it make more sense to divide the focus groups into those that are higher achieving and those that are not and see if the higher achieving group uses deliberate practice more often? That would support the authors' assertion that deliberate practice is helpful to the learning process.

- **We decided to divide focus groups by PGY level to determine if deliberate practice was utilized more as the resident progressed through their program, which developed in the findings (i.e., the difference between juniors and seniors in ways that they prepare, execute, and monitor feedback). As researchers, we were not able to determine who of the participants were “high achieving” prior to data collection.**

6. Why collect/report gender information if it has nothing to do with the stated purpose of the study?

- **Although not related to the stated purpose of the study, gender was included to give readers an understanding of the composition of each focus group.**

7. The first sentence in the section "Data collection" does not make sense.

- **The first sentence was revised to: A semi-structured interview guide was developed** **in accordance to the theoretical framework of deliberate practice ^3^ by the second author (M.D.).**

8. The questions asked of the focus group participants feel like they are leading. For example, asking "how did you prepare/plan for the surgical case" assumes that the learner did some sort of preparation/planning. It would be less leading to ask "did you prepare/plan for the surgical case" and then explore further if the answer is "yes".

- **This is a great point, however, the interview guide was based on the deliberate practice framework with the CBME principles in minds. Therefore, questions were developed to encompass these elements and the expectation that surgical residents practice these techniques regularly.**

9. How did the authors decide which questions to ask during the qualitative interview? Do any of the authors have advanced training on qualitative interviewing and analysis of qualitative data?

- **All questions from the interview guide (** **https://doi.org/10.6084/m9.figshare.21346830.v1** **) were asked during focus groups with the addition of some probing questions. Both authors are trained qualitative researchers, with the first author being a qualitative research expert. Both authors qualitative experience has been added to the Data Analysis section.**

​​​​​​​10. Discussion - Although the ultimate goal is to improve patient outcomes, the authors did not study this so they should get rid of the phrase "...and in turn improving patient outcomes" at the end of the first paragraph.

- **This sentence has been removed. **
- **In addition to your feedback and suggestions, I have removed the section on future research from the discussion and have added the suggestions to the appropriate paragraphs throughout the discussion for better readability and purpose. **
